# 

**Published:** 1873-09

**Authors:** 


### Books and Pamphlets Received.

Clinical Electro-Therapeutics, Medical and Surgical. A Hand-Book for Physicians in the Treatment of Nervous and other Diseases. By Allan McLane Hamilton, M. D. New York: D. Appleton & Co., 1873. Buffalo: Martin Taylor.

The Cerebral Convulutions of Man, represented according to original observations, especially upon their development in the Fœtus, intended for the use of Physicians. By Alexander Ecker, Prof. of Anatomy, University of Freiburg, Baden. Translated by Robert T. Edes, M. D. New York: D. Appleton & Co., 1873. Buffalo: Martin Taylor.

Insanity in its Relations to Crime. A Text and a Commentary. By Wm. A. Hammond, M. D. New York: D. Appleton & Co., 1873.

Report upon some of the recent Researches in Neuropathology. By Wm. B. Neftel, M. D. Reprint from No. 5 Archives of Scientific and Practical Medicine.

Clinical Notes on Nervous Diseases of Women. By Wm. B. Neftel, M. D. Reprint from Nos. 3, 4, and 5 Archives of Scientific and Practical Medicine.

Spectrum Analysis of Old Blood Stains. By S. Watermrn, M. D. Reprint from No. 5, Archives of Scientific and Practical Medicine.

The Effects of High Atmospheric Pressure, including the Caisson Disease. By Andrew H. Smith, M. D., Surgeon to N. Y. Bridge Company.

The Proper Treatment of Children, Medical or Medicinal. Being the Annual Discourse before the Massachusetts Medical Society, June 4th, 1873. By Charles E. Buckingham, M. D.

Infant Feeding and its Relation to Infant Mortality. Reprint from New York Medical Review.

Transactions of the Medical Society of the State of West Virginia, June 1873.

### Medical and Surgical Journal

PUBLISHED MONTHLY,

Containing Original Articles, Reports of Medical Societies and Hospitals. Editorial, Reviews, Correspondence, Army News, etc., etc.; including the usual variety of Medical Periodical Publications. Specimen copies sent on application.

Address Buffalo Medical & Surgical Journal, Buffalo, N. Y.

TERMS OF SUBSCRIPTION, - - $3.00 PER YEAR, IN ADVANCE.

